# Low‐coverage reduced representation sequencing reveals subtle within‐island genetic structure in Aldabra giant tortoises

**DOI:** 10.1002/ece3.8739

**Published:** 2022-03-18

**Authors:** F. Gözde Çilingir, Dennis Hansen, Nancy Bunbury, Erik Postma, Richard Baxter, Lindsay Turnbull, Arpat Ozgul, Christine Grossen

**Affiliations:** ^1^ 27217 Department of Evolutionary Biology and Environmental Studies University of Zurich Zurich Switzerland; ^2^ Zoological Museum University of Zurich Zurich Switzerland; ^3^ Indian Ocean Tortoise Alliance Victoria Seychelles; ^4^ Seychelles Islands Foundation Victoria Seychelles; ^5^ Centre for Ecology and Conservation College of Life and Environmental Sciences University of Exeter Penryn UK; ^6^ Plant Sciences Department University of Oxford Oxford UK

**Keywords:** *Aldabrachelys gigantea*, conservation genomics, ddRAD‐seq, genotype likelihoods, giant tortoises, low‐coverage sequencing

## Abstract

*Aldabrachelys gigantea* (Aldabra giant tortoise) is one of only two giant tortoise species left in the world and survives as a single wild population of over 100,000 individuals on Aldabra Atoll, Seychelles. Despite this large current population size, the species faces an uncertain future because of its extremely restricted distribution range and high vulnerability to the projected consequences of climate change. Captive‐bred *A*. *gigantea* are increasingly used in rewilding programs across the region, where they are introduced to replace extinct giant tortoises in an attempt to functionally resurrect degraded island ecosystems. However, there has been little consideration of the current levels of genetic variation and differentiation within and among the islands on Aldabra. As previous microsatellite studies were inconclusive, we combined low‐coverage and double‐digest restriction‐associated DNA (ddRAD) sequencing to analyze samples from 33 tortoises (11 from each main island). Using 5426 variant sites within the tortoise genome, we detected *patterns* of within‐island population structure, but no differentiation between the islands. These unexpected results highlight the importance of using genome‐wide genetic markers to capture higher‐resolution genetic structure to inform future management plans, even in a seemingly panmictic population. We show that low‐coverage ddRAD sequencing provides an affordable alternative approach to conservation genomic projects of non‐model species with large genomes.

## Graphical abstract

The Aldabra giant tortoise is one of only two giant tortoise species left in the world and survives as a single wild population on Aldabra Atoll, Seychelles. We combined low‐coverage and double‐digest restriction‐associated DNA (ddRAD) sequencing and detected patterns of within‐island population structure, but no differentiation between the islands. These unexpected results highlight the importance of using genome‐wide genetic markers to capture higher‐resolution genetic structure to inform future management plans, even in a seemingly panmictic population.

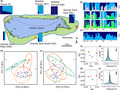

## 1 INTRODUCTION

Many endangered species are restricted to a single or a small number of remnant populations. Management efforts often include introductions from these source populations to other suitable locations to lessen the risk of extinction or because the species in question are ecosystem engineers and can be used to restore degraded habitats elsewhere. However, such interventions have important implications for the genetic future of the newly founded population. As only a subset of the individuals in the source population can be moved, genetic diversity is at risk to be lost and artificial population structure may be created in the new populations. Genetic diversity is essential for the adaptive potential of a species, particularly in the face of environmental changes and disease outbreaks (Reed, 2005; Reed & Frankham, 2003). Hence, management decisions need to be carefully planned to take the genetic characteristics of the source populations into account to aim at retaining as much genetic diversity as possible (Hoban et al., 2021).

One problem with assessing current genetic characteristics of endangered non‐model species is that suitable marker systems, such as simple sets of microsatellites, are often unavailable. Next‐generation sequencing provides promising tools at decreasing costs (Davey & Blaxter, 2010; Hayden, 2014). However, it can still be financially overwhelming and (if not outsourced) bioinformatically challenging to generate high‐quality whole genomes, especially for species with large genomes, and because more than a handful of sequenced individuals are needed for population genomics studies (Corlett, 2017; Shafer et al., 2015). One potential solution is to use reduced representation sequencing, such as restriction‐associated DNA (RAD) sequencing, which does not require a reference genome and is generally cost‐effective (Andrews et al., 2016; Davey & Blaxter, 2010). Financial and computational costs of whole‐genome sequencing of many individuals can be further reduced by adopting a low‐depth sequencing strategy (Pasaniuc et al., 2012), where information on the whole genome is obtained, but at low coverage (generally 1–2×). This approach risks loss of genotype accuracy, which can be overcome by inferring genotype likelihoods (Fumagalli et al., 2014; Korneliussen et al., 2014). This genotype‐free estimation of allele frequencies has been shown to reduce biases and improve demographic inference from RAD‐seq data (Warmuth & Ellegren, 2019). Interestingly, to date, only a small number of studies have combined RAD and genotype‐free estimation of allele frequency estimation approaches (Bay et al., 2019; Breusing et al., 2019; Peart et al., 2020; Záveská et al., 2019).

**FIGURE 1:**
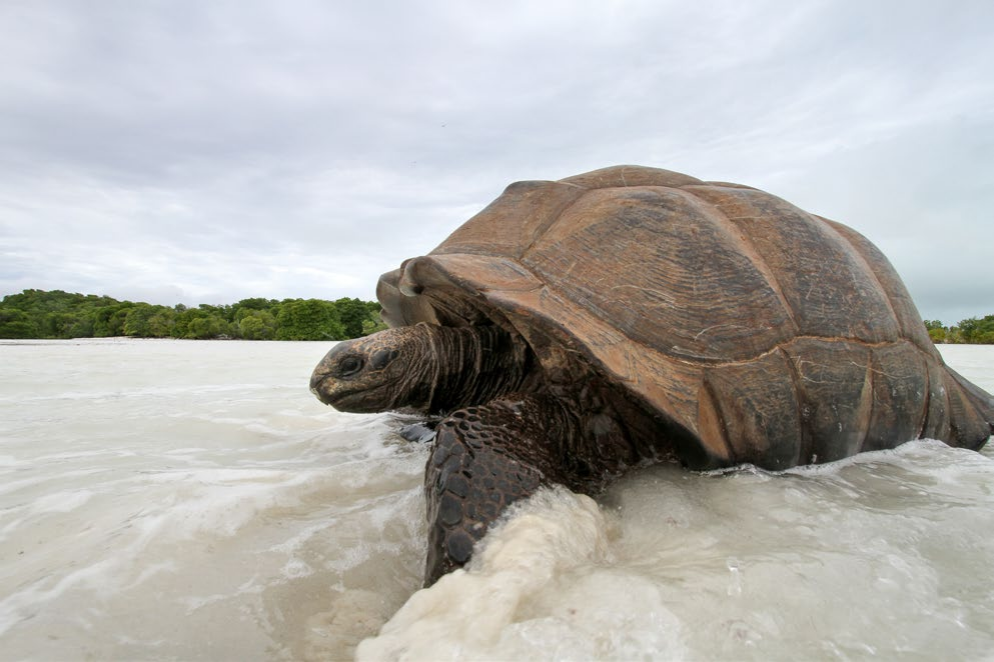
An Aldabra giant tortoise entering the Aldabra Lagoon

Here, we use low‐coverage ddRAD sequencing as a time‐ and cost‐effective approach for the population genetic analysis of *Aldabrachelys gigantea*, Schweigger 1812 (Aldabra giant tortoise) (Figure 1), a flagship and keystone species lacking both a suitable marker set and a reference genome. *Aldabrachelys gigantea* is one of only two giant tortoise species left in the world together with the Galápagos giant tortoise complex, *Chelonoidis niger* (Turtle Taxonomy Working Group, 2017), and is endemic to Aldabra Atoll, Seychelles. The species is currently listed as Vulnerable by the IUCN Red List (version 2.3) due to its limited distribution in the wild and threats posed by climate change. It is the only survivor of at least nine endemic giant tortoise species that once lived on Western Indian Ocean islands (Austin et al., 2003; Palkovacs et al., 2002) and occupies a prominent functional role in shaping and sustaining large‐scale vegetation dynamics as it is the largest frugivore and herbivore in its island ecosystem (Hansen, 2015; Hnatiuk et al., 1976; Merton et al., 1976). Therefore, *A*. *gigantea* are currently used to help restore degraded native ecosystems on several other Western Indian Ocean Islands (Griffiths et al., 2011, 2013; Hansen et al., 2010).

*Aldabrachelys gigantea* was on the verge of extinction in the late 19th century due to excessive harvesting, with a population low in around 1870 of somewhere between <1000 and a few thousand tortoises (Bourn et al., 1999; Stoddart & Peake, 1979). Thanks to calls for protection from Charles Darwin and others in 1874, the number of *A*. *gigantea* increased quickly to several tens of thousands in the 1960s to today's stable population of well over 100,000 individuals (Turnbull et al., 2015). The Aldabra population is divided into several subpopulations across the different islands that make up the atoll.

Two previously published genetic studies of *A*. *gigantea* have involved samples from Aldabra's wild population and were all based on the mitochondrial control region or microsatellite data. The first, by Palkovacs et al. (2002), focused on captive individuals and examined potential genetic differentiation between morphotypes. Although their sampling included some wild individuals, they did not examine the population structure within the atoll. The second study (Balmer et al., 2011) was based on samples from Malabar, Grande Terre South, and Grande Terre East. They found strong genetic differentiation between the two Grande Terre localities, and between Malabar and Grande Terre. They concluded that movements between different areas and islands are rare. Their sampling did not include samples from Picard and the study relied on eight microsatellite markers originally designed for *Chelonoidis niger* (split 35–40 mya, Kehlmaier et al., 2019). Using molecular markers developed for other species bears the risk of ascertainment bias and an underestimation of genetic variation (Delport et al., 2006; Ellegren et al., 1995). Similar problems have been encountered in other microsatellite studies of, for instance, turtles (Çilingir et al., 2019), fish (Carreras et al., 2017), and mammals (Hendricks et al., 2017; Mesnick et al., 2011).

Here, we provide a new sampling scheme for the first time including all the main islands hosting Aldabra giant tortoises and a new analysis that acts as a case study for the conservation genetic analysis of a non‐model species using low‐coverage sequencing combined with double‐digest restriction site‐associated DNA sequencing (ddRADseq; Peterson et al., 2012). Our specific aims are as follows:

1. To quantify the overall genetic structure of the endemic *A. gigantea* population
2. To determine whether there are significant differences in the genetic composition of the species among and within islands.

## 2 MATERIALS AND METHODS

### 2.1 Study system

**FIGURE 2:**
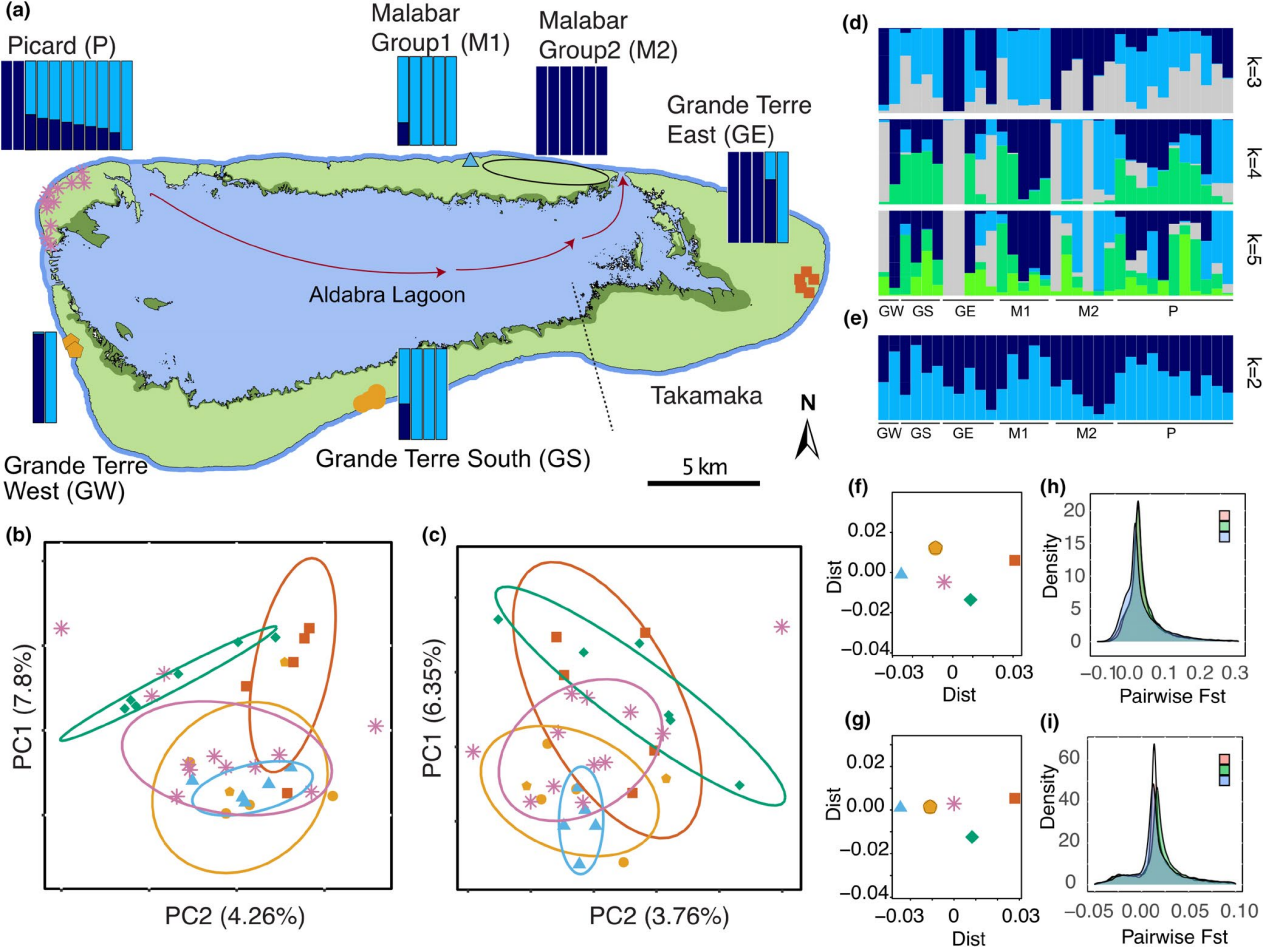
(a) Aldabra Atoll's four main islands. The curved arrow within the map indicates the direction of the ocean currents. Darker green shaded areas show the mangrove distribution within the atoll. Dashed lines show the region of Takamaka. Every colored mark on the map represents a sampled tortoise. The shape of the marks indicates a distinct sampling location within each island. The exact sampling location of only one Malabar individual is known. The area delimited by a black ellipse shows approximate sampling locations of all remaining Malabar samples on the northeastern side of the island. Each bar above the islands corresponds to one individual sampled there and shows its admixture proportions estimated with the main dataset assuming two ancestral populations (*k* = 2). Light blue bars: cluster A, Dark blue bars: cluster B. (b) Five genetic clusters are shown on the PCA plot of the main dataset and (c) the downsampled dataset. Every colored mark represents an individual. Malabar Group 2 individuals are shown with green diamonds. (d) Admixture proportions of all the individuals estimated with the main dataset assuming *k* = 3–5, (e) with downsampled dataset assuming *k* = 2. (f) MDS (multidimensional scaling) graph of the pairwise *F*_ST_ values estimated for each group with the main dataset. (g) MDS graph of the downsampled dataset, each mark represents the whole group. (h) Density plot of the sliding window analysis of pairwise *F*_ST_ between three genetic groups representing within‐ and among‐island genetic differentiation, estimated with the main dataset (GE‐GS&W and M1‐M2, within Grande Terre and Malabar, respectively; GE‐M2, among Grande Terre and Malabar) and (i) estimated with the downsampled dataset

The endemic distribution of *A*. *gigantea* is restricted to Aldabra Atoll, in the southern Seychelles. The atoll consists of four main islands, Grande Terre, Malabar, Polymnie, and Picard (Figure 2a), separated by channels and enclosing a shallow lagoon. On the atoll, giant tortoises are unevenly distributed across the three largest islands (Polymnie, the smallest main island, has no tortoises) due to environmental differences (e.g., terrain, food, freshwater resources, and shade availability) and differences in exploitation history (Bourn & Coe, 1978; Turnbull et al., 2015; Walton et al., 2019). Effective conservation management measures saving the species from extinction in the late 19th century included the reintroduction of tortoises to Picard and atoll‐wide invasive species control (Bourn et al., 1999; Bunbury et al., 2018; Stoddart & Peake, 1979; Turnbull et al., 2015). The largest population lives on Grande Terre, with the second largest on Malabar. Polymnie, surrounded by deep channels, remains empty of *A*. *gigantea*, while Picard has been repopulated in several translocations of tortoises from Malabar and Grande Terre since the early 1900s, with the last occurring in the 1980s. An unknown number of *A*. *gigantea* occur around the globe in captivity, semi‐natural, or rewilded populations (Hansen et al., 2010).

### 2.2 Sample collection and DNA extraction

In 2012 and 2013, approximately 100 µl of blood were drawn from the cephalic vein of an extended front limb of 33 adult *A*. *gigantea* individuals representing the three main islands of Aldabra, which are inhabited by tortoises: 11 from Picard, 11 from eastern Malabar, and 11 from Grande Terre (West, *n* = 2; South, *n* = 4; and East, *n* = 5) (Figure 2a, Table S1). Absolute ethanol was added to the blood samples in a 1:20 ratio to prevent coagulation (Wietlisbach, 2017). All samples were stored at room temperature until arrival in the lab and then at −80°C until DNA extraction.

DNA extraction was performed with 3 µl of blood (in ethanol) per sample, using the sbeadex™ kit (LGC Genomics, Middlesex, UK), following the manufacturer's protocol for DNA extraction from nucleated red blood cells. Genomic DNA concentrations were measured with a dsDNA Broad Range Assay kit (Qubit 2.0 Fluorometer, Invitrogen, Carlsbad).

### 2.3 ddRAD‐seq library preparation and sequencing

To keep sequencing costs as low as possible, we used a reduced representation genome sequencing approach, specifically the double‐digest restriction site‐associated DNA sequencing (ddRAD‐seq, Peterson et al., 2012). Restriction enzymes were selected based on in silico double‐digest runs, using the SimRAD package within R v4.0.3 (Lepais & Weir, 2014; R Core Team, 2020). Enzyme combinations of EcorI‐TaqI, EcoRI‐MspI, and EcoRI‐BfaI were tested using *in silico* restriction digests, performed on the basis of a *Chelonoidis abingdonii* (Galápagos giant tortoise) genome (NCBI BioProject PRJNA611832), which is the phylogenetically closest available genome for *A*. *gigantea* (ca. 35–40 M.Y. of divergence time; Kehlmaier et al., 2019; Quesada et al., 2019). We aimed for approximately 50,000 *in silico* RAD loci, which was achieved with the selected enzyme combination EcoRI‐BfaI with a target size selection window of 300–350 bp (52,000 expected ddRAD loci, Figure S1).

We used 100 ng genomic DNA from each sample (*n* = 33) for the digestion. A single ddRAD‐seq library was prepared by processing the 33 samples following the protocol by Peterson et al. (2012) with slight modifications as described in Çilingir et al. (2021). Briefly, after double digestion, the products were cleaned with a 1.0× ratio of AMPure XP beads. Next, the P1 adapters containing the inline barcodes unique to each sample (Peterson et al., 2012), and with an EcoRI overhang and the P2 adapter with a BfaI overhang, were ligated to the restricted DNA. Then, equal amounts of individually barcoded DNA were pooled. The double size selection was performed with a total of 300 μl pooled aliquot by treatment with 0.5× and 0.12× AMPure XP beads. After the size selection, eight PCR cycles were run using the common PCR1 and the PCR2 primers, which include a standard Illumina index (Peterson et al., 2012). In our case, only one index was used as there was only one sequencing library prepared. A final AMPure XP beads clean‐up was followed with a 0.6× bead ratio. The quality check of the final library fragment size range was performed with a Bioanalyzer High Sensitivity DNA kit (Agilent, Santa Clara, CA). Finally, 10 picomoles of the quality‐checked library were sequenced on an Illumina Miseq platform for a paired‐end run on one lane at the Genetic Diversity Center, ETH Zurich, Switzerland, yielding paired‐end read lengths of 300 bp each.

Data quality of the sequences was assessed using FastQC v0.11.9 (S. Andrews, 2010), and the adaptor sequences of the Illumina sequencing platform were trimmed using Trimmomatic v0.39 (Bolger et al., 2014) (ILLUMINACLIP:2:30:10:2). Adapter‐trimmed data were demultiplexed in Stacks v2.53 (Rochette et al., 2019) using process_radtags and allowing one barcode mismatch. At this step also all reads containing at least one N (uncalled base) were removed. Quality filtering of the demultiplexed data was done with Trimmomatic (Bolger et al., 2014) requiring an average Phred quality score per entire read of at least 20 (AVGQUAL:20), an average quality of 10 in a sliding window of 30 before cutting the read (SLIDINGWINDOW:30:10), bases were cut off the end of the read if the quality dropped below 19 (TRAILING:19), and the first 10 bases were cropped to remove the enzyme cut sequence (HEADCROP:10).

### 2.4 Alignment to a reference genome, estimation of sequencing depth, and downsampling

After quality filtering, the paired reads were aligned to the *C*. *abingdonii* reference genome using BWA‐MEM version 0.7.17 (Li & Durbin, 2009). Calculation of the average per site sequencing depth for each individual was done in three following steps. First, SAMtools (Li et al., 2009) was used to extract properly paired reads with mapping quality of >20 from the BAM file of individual GrdTr_11 (the individual with the highest number of sequence reads, Table S1). Next, for each individual, all positions with at least one read were retained within a bed file by using bedtools v2.29.2 (Quinlan & Hall, 2010). Subsequently, per‐site sequencing depth per individual was calculated using SAMtools (Li et al., 2009) based on the range given by the bed file (all sites with at least 1× coverage).

Because the average sequencing depth per individual varied considerably, we repeated the major analyses after downsampling the forward and reverse Fastq files of each sample to equalize the number of reads per individual with seqtk v1.3 (https://github.com/lh3/seqtk) to 154,599 reads (number of reads of individual Picard_2, third‐lowest read count, Table S1).

### 2.5 Estimation of genotype likelihoods

As the mean sequence coverage per sample was low (2.28×; range: 0.2–6.1×, Table S1), the uncertainty of genotypes was accounted for in the subsequent analyses by computing the genotype likelihoods at variant sites instead of calling genotypes. Accordingly, the read alignments of all 33 individuals were processed with ANGSD v0.93 (Korneliussen et al., 2014), a software developed for genomic analyses of low‐coverage data. The GATK (Genome Analysis Toolkit) model was used (McKenna et al., 2010), and major and minor alleles were directly inferred from the genotype likelihoods (doMajorMinor 1, doMaf 1). Quality filtering for the subsequent downstream analyses was performed as follows: Only properly paired (only_proper_pairs 1) and unique reads (uniquieOnly 1) were used, and only biallelic sites were retained (skipTrialleleic 1). Nucleotides with base qualities lower than 20 were discarded. Excess of SNPs around indels and excessive mismatches with the reference were corrected by realignment (C50, baq 1 [Li, 2011]). Reads with a mapping quality lower than 20 were discarded.

Additionally, for the estimation of genotype likelihoods, only SNPs with a *p*‐value <10^−6^ (the significance threshold for polymorphism detection) and heterozygosity <0.5 were retained, the latter to exclude potential paralogs (Hardy, 1908; Hohenlohe et al., 2011). Further filters were applied depending on the analysis. For the population genetic structure analyses, sites with read data in fewer than 30 of the 33 samples were excluded (minimum representation among samples >90%, ‐minInd 30). The minimum depth of sites to be retained was also set to 30, and hence, on average, at least one read per individual was required. The maximum depth per site was set as the sum of the average sequencing depth and two times the standard deviation (373 for the main dataset, 128 for the downsampled dataset). For the estimation of genetic differentiation and diversity, which were calculated per group, at least 50% of the samples in a particular group had to be represented (minInd = 50% of all individuals in a group). The minimum depth for each group was set to the minimum number of individuals allowed (50% of the overall individuals within a group) and the maximum depth was the average plus two times the standard deviation for each group.

### 2.6 Estimation of kinship

To check for possible familial relationships potentially affecting the population structure analyses, the coefficient of kinship (Jacquard, 1974) was inferred by using NgsRelate v2 (Hanghøj et al., 2019). To achieve this, allele frequencies and genotype likelihoods estimated with the main dataset were used and average coefficients of kinship for all possible individual pairs were calculated.

### 2.7 Population genetic structure

For a first overview of the population structure, a principal component analysis was carried out with PCAngsd v09.85 (Meisner & Albrechtsen, 2018) with an additional minor allele frequency (MAF) filter of 0.01 or 0.05. As a complementary population structure analysis, we used the clustering tool NGSAdmix (Meisner & Albrechtsen, 2018; Skotte et al., 2013). Similar to the Bayesian clustering method STRUCTURE (Pritchard et al., 2000), NGSAdmix allows the estimation of individual admixture proportions by assigning individuals to different clusters. While a PCA allows the assumption‐free visualization of the genetic relatedness among individuals, NGSAdmix tries to minimize the within‐group variation to define genetic groups and estimate individual admixture proportions (Meisner & Albrechtsen, 2018; Skotte et al., 2013). To use NGSAdmix, it is recommended to perform LD pruning (i.e., to filter sites based on pairwise linkage disequilibria) as the program assumes the independence of genomic loci (Skotte et al., 2013). Hence, pairwise linkage disequilibria (LD) were calculated using ngsLD (Fox et al., 2019) and LD pruning was performed by allowing a maximum among SNP distance of 100 kilobases and a minimum weight of 0.5. A total of 100 replicates were performed for each NGSAdmix run and the number of clusters (*k*) varied between 2 and 10. The results were analyzed and visualized with CLUMPAK (Kopelman et al., 2015), and the log‐likelihoods calculated for each run were visualized in R (R Core Team, 2020).

### 2.8 Estimation of genetic differentiation and diversity comparison

As a measure of population differentiation, weighted pairwise *F*_ST_ values were calculated between each group of three different islands (Picard, Malabar, and Grande Terre) and each group based on our population structure analyses (total of five groups on three islands: Malabar‐1, Malabar‐2, Grande Terre East, Grande Terre South & West, and Picard; see also Figure 2) by using ANGSD (Korneliussen et al., 2014) and realSFS (a module of ANGSD). For each group, the site allele frequency (SAF) likelihoods were estimated based on individual genotype likelihoods (see section Estimation of Genotype Likelihoods) with the ‐doSAF 1 option of ANGSD (Korneliussen et al., 2014). The SAF was polarized with the reference genome as no ancestral sequences were available. Then, folded site frequency spectra (SFS) were calculated for each population and *F*_ST_ metrics were estimated using 2D‐SFS and the option ‐whichFst 1. To visualize the genetic differentiation between all groups/populations, multidimensional scaling (MDS) was applied to the pairwise *F*_ST_ matrix using the cmdscale function in R (R Core Team, 2020). Moreover, a heatmap of the pairwise *F*_ST_ values was generated with ggplot2 (Wickham, 2016) in R (R Core Team, 2020). Additionally, to account for potential local effects along the genome, a sliding window analysis of the pairwise *F*_ST_ values was performed for the comparison within Malabar and Grande Terre Islands, as well as among Grande Terre and Malabar with a window and step size of 50 kilobases (non‐overlapping windows, excluding windows with <10 sites).

Possible differences in genetic diversity among the five groups defined above, Malabar‐1, Malabar‐2, Grande Terre East, Grande Terre South & West, and Picard (see also Figure 2), were investigated by calculating average number of pairwise differences or nucleotide diversity (*π*; [Tajima, 1989]) and population mutation rate (Watterson's *θ*; [Watterson, 1975]). Both measures were based on SFS estimates and performed with the realSFS and ThetaStat modules in ANGSD (Korneliussen et al., 2014). Estimates of Watterson's *θ* and π were obtained per genome region via a sliding window analysis with a window and step size of 10 kilobases (non‐overlapping windows, excluding windows with <10 sites). A Tukey's range test (David & Tukey, 1977) was applied to compare the diversity measures among different groups. Since Tukey's range test is a post hoc test, initially ANOVA was performed on the data.

## 3 RESULTS

### 3.1 Genotype likelihood analysis

The sequencing effort yielded 23,517,270 raw reads for a total of 33 samples. After adapter removal, quality checking, and demultiplexing, an average of 1,188,685 (range: 129,188–2,610,656, see also Table S1) reads per sample were retained in the main dataset. The mean mapping rate was 96.5% (range: 94.7–97.0%, Table S1), resulting in a mean sequencing depth per sample of 2.28× (range: 0.2–6.1×, Table S1). In the downsampled dataset, all individual fastq files were downsampled to 154,599 sequencing reads and the mean sequencing depth per sample was calculated as 0.56× (range: 0.2–0.8×). The genotype likelihood analysis with ANGSD (Korneliussen et al., 2014) resulted in 238,995,840 sites, 6153 of which were retained as variant sites (SNPs). MAF filtering for >0.05 yielded 5426 SNPs. A total of 189,369,839 sites were obtained using the downsampled dataset, 1755 of which were retained as variant sites (SNPs). MAF filtering for >0.05 yielded 1632 SNPs, all of which were also found among the SNP set of MAF>0.05 obtained with the main dataset.

### 3.2 Population genetic structure

We were primarily interested in the overall genetic structure and differentiation among islands. The PCA of the main dataset revealed two distinct clusters (PC1:7.8% and PC2: 4.26%, Figure 2b). One cluster was represented by all Grande Terre South (GS) individuals, one individual from Grande Terre West (GW), five individuals from Malabar (now termed “Malabar Group 1” or M1), one Grande Terre East (GE) individual, and the majority of all Picard (P) individuals (*n* = 9). The second cluster grouped four of the five GE individuals, six Malabar individuals (now termed “Malabar Group 2” or M2), one GW individual, and the two remaining P individuals (Figure 2b). M1 was clearly separated from M2, and GS, GW, and GE formed overlapping but differing groups. P individuals overlapped with all remaining groups. The PCA of the downsampled dataset (PC1: 6.35% and PC2:3.76% Figure 2c) resulted in a less clear resolution but confirmed the general population structure described above for the full dataset. Changing the minor allele cut‐off (MAF > 0.01 vs. MAF > 0.05) had no effect on the PCA structure (Figure S2A,B).

Next, we wanted to investigate if the observed population structure is consistent with two genetic groups as indicated by the PCA and we aimed at estimating admixture proportions. For this analysis, a total of 3781 LD‐pruned SNPs with MAF >0.05 were used. The admixture proportions indicated that when the number of putative clusters was assumed to be 2 (k=2 was the most likely number of k based on log likelihoods, Figure S3A), the M1 individuals were assigned to cluster A and the M2 individuals were assigned to cluster B, except for one individual of M1, which showed mixed ancestry (Figure 2a). Also, Grande Terre showed high within‐island differentiation with most of GE and one GW individual assigned to cluster B and most of GS and the remaining GW individual to cluster A. All P individuals except for two assigned to cluster B showed mixed ancestry. Hence, genetic groups of different islands were assigned to the same clusters (M1 with GS&W, and M2 with GE). This high within‐island differentiation on Malabar and Grande Terre but lower among‐island differentiation confirmed the outcome from the PCA (Figure 2b). Under a scenario of 3–10 hypothetical clusters, all groups showed mixed ancestry (Figure 2d, Figure S4). The results of the admixture analysis based on the downsampled dataset including a total of 1120 LD‐pruned SNPs with MAF >0.05 were consistent with the results based on the main dataset (Figure 2e; Figures S3B, S5 and S6). Admixture proportions obtained with the two datasets were positively correlated, but the retained resolution was considerably lower (Figure 2e, Figure S6).

The average coefficient of kinship for all possible individual pairs was 0.01 within sampling locality (range: 0–0.08) and 0.008 (range: 0–0.17) among sampling localities (Table S1B). Estimates for GE, GS&W, M1, M2, and P were 0.013 (range: 0–0.08), 0.008 (range: 0–0.02), 0.005 (range: 0–0.05), 0.021 (range: 0–0.04), and 0.006 (range: 0–0.03), respectively (Table S1B). Hence, there is no evidence for potential familial structure within sampling localities explaining the observed population structure.

### 3.3 Estimation of genetic differentiation and summary statistics

**FIGURE 3:**
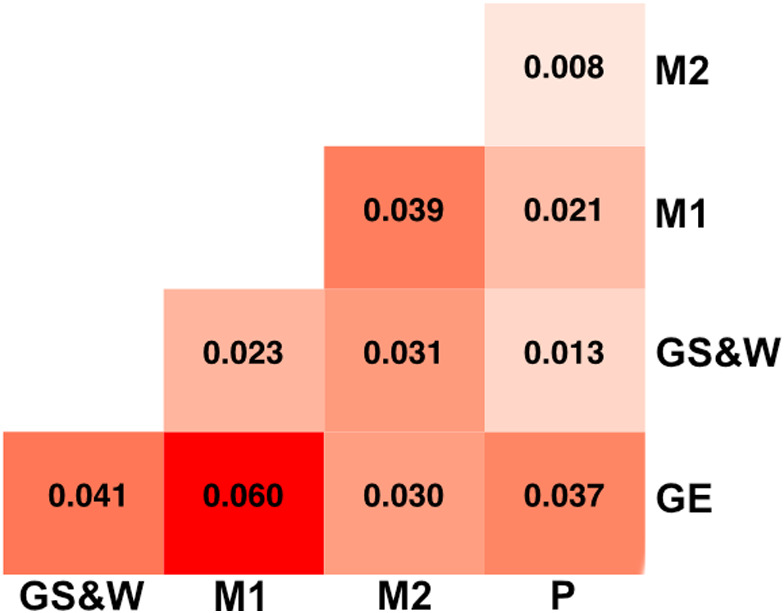
Heatmap of pairwise genetic differentiation (measured as *F*_ST_), estimated for five different locations using the main dataset

All pairwise *F*_ST_ estimates calculated among the three Aldabra Islands in the study (Picard, Malabar, and Grande Terre) were 0, suggesting no evidence for among‐island differentiation. As expected from the PCA and the admixture proportion analysis, the major differentiation was found between M1 and GE (0.06), followed by GE and S&W (0.041) and M1 and M2 (0.039) (Figures 2f and 3). To account for possible local effects along the genome, we also compared within‐ and among‐island differentiation of Grande Terre and Malabar by performing a sliding window analysis of pairwise *F*_ST_ values. The analysis confirmed a slightly lower among‐island differentiation between Malabar and Grande Terre than within‐island differentiation on Malabar. The analysis of the main dataset indicated a level of within‐island differentiation on Grande Terre similar to that on Malabar, but this differentiation was lower when analyzing the downsampled dataset (Figures 1i and 2h). The pairwise *F*_ST_ estimations with the downsampled dataset confirmed the major finding of within‐island differentiation (Table S2, Figure 2).

**FIGURE 4:**
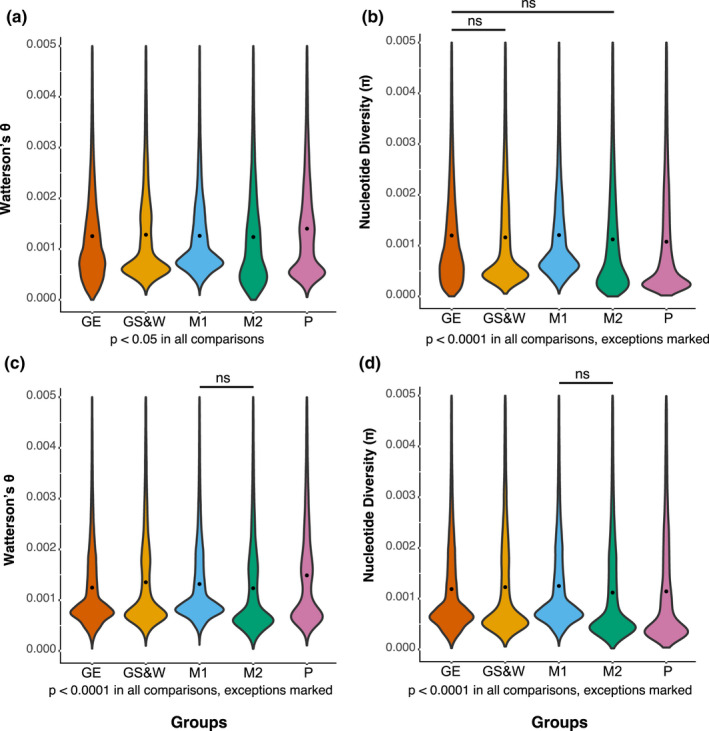
(a) Per‐site estimates of Watterson's *θ* (b) and nucleotide diversity (*π*) obtained via a sliding window analysis performed with the main dataset; (c) per‐site estimates of Watterson's *θ* (d) and nucleotide diversity (*π*) obtained via a sliding window analysis performed with the downsampled dataset. Each group is colored the same as in Figure 2 (orange, Grande Terre East; yellow, Grande Terre South & West; blue, Malabar Group 1; and green Malabar Group 2; pink, Picard) and the average value per each group is indicated with a black dot

Mean Watterson's *θ* values of all the groups ranged from 0.00139 to 0.00167, with P having the highest estimate and GE the lowest (Figure 4a), suggesting highest genetic diversity in P. There was significant variation among the groups, *F* (4, 596387) = 459, *p* < 2e−16. All the groups’ mean Watterson's θ values were significantly different from each other at *p* < .05. Mean π per group was 0.00143–0.00153, with P having the highest and M2 the lowest values (Figure 4b), again suggesting highest genetic diversity in P. Although the absolute differences among groups were small, there was significant variation among the groups, *F* (4, 596387) = 75.23, *p* < 2e−16. Mean π values of all the groups differed from each other, except for GE, which did not differ from M2 or GS&W. While both Wattersons *θ* and *π* as well the analyses with the downsampled dataset suggested highest diversity in P, differences in diversity among the other groups were small (but significant) and the relative order of groups differed between analyses.

## 4 DISCUSSION

We used low‐coverage RAD sequencing to investigate the population genetic structure and variation in the endemic *A*. *gigantea* population. Our data, although relying on a relatively small sample size (5–11 per island/sampling location), not only revealed the subtle genetic structure of previously bottlenecked populations but also suggested a potentially greater role of passive movement between islands via water in a terrestrial species than previously expected.

Our study is one of few to focus on a combination of reduced representation sequencing and the genotype likelihood approach to study the population genomics of a non‐commercial and non‐model species. Our case study supports the use of low‐coverage ddRAD sequencing instead of the low‐coverage whole‐genome sequencing (Lou et al., 2021), which is still costly for large genomes and/or sample sizes. Sequencing costs depend on the platform, but could be as low as 5.25 USD per sample using our approach (2–3× coverage or 0.3Gb). In contrast, a low‐coverage whole‐genome resequencing project for a genome of about 2.4 Gb (the approximate genome size of *A*. *gigantea*) would result in sequencing costs of about 105 USD per sample (2–3× coverage or 6 Gb). Our reduced representation approach could therefore be particularly useful for species with very large genomes.

We also investigated the effects of unevenly distributed depth of sequencing per individual by repeating all analyses with a downsampled dataset. We showed that the results obtained with both datasets were consistent, but the downsampling led to a loss of resolution, especially for the admixture analysis. The smaller number of loci and among‐locus variation in coverage known for RAD (Davey et al., 2013; O’Leary et al., 2018) may mean that there is a minimum acceptable depth of coverage for this technique.

### 4.1 Unexpected partitioning of genetic structure

We found lower among‐island than within‐island differentiation. Specifically, our analysis suggested two main groups of genetic variation (Figure 2a): M2, and all but one individual from GE represented an eastern group; and M1 and all but one individual from GS&W represented a western group. The P individuals were assigned to both clusters, which was expected, given that the original population of Picard was extirpated in the 1800s, and the current population originates from reintroduced tortoises from Grande Terre and Malabar (Bourn et al., 1999). These findings were supported by the PCA and the pairwise *F*_ST_ analyses, which showed minor differentiation between Picard and the other islands, but stronger differentiation within Grande Terre and Malabar. The genetic differentiation between GE and GS&W suggests that connectivity along the east–west axis of the island may be limited. This is in agreement with behavioral, ecological, and geographic observations (Bourn & Coe, 1978; Gibson & Hamilton, 1984; Swingland et al., 1989), and a previous study by Balmer et al. (2011). Areas of thick *Pemphis* scrub and deeply fissured rocks appear to limit the movement of tortoises (Gibson & Hamilton, 1984). Hence, geographical barriers such as the region around Takamaka (dotted line in Figure 2a) that include deeply fissured limestone and thick *Pemphis* scrub together with isolation by distance probably explain the observed substructure on Grande Terre already described by Balmer et al. (2011) (see also Bourn & Coe, 1978).

More surprising and different from the previous findings of Balmer et al. (2011) was the low differentiation between M2 and GE. Occasional movement of tortoises carried by tidal currents from the mangrove area in Grande Terre East to the coastal area of M2 may cause inter‐island gene flow (Figure 2a). The tortoises often spend days or weeks in the muddy mangroves of Grande Terre East. Sometimes they move against tidal waters rushing out or in, with a risk of being swept away, and tortoises can even be spotted adrift in the open ocean outside the reef (Hansen et al., 2017). Ocean currents are increasingly acknowledged for their importance in shaping population structure (Arjona et al., 2020; White et al., 2010) and also for terrestrial reptiles (Calsbeek & Smith, 2003; Hawlitschek et al., 2017). The movement of animals by humans could also explain the low differentiation. Although it is known that animals were transported from Grande Terre and Malabar to Picard for conservation purposes, there is no record of animals being transported from Grande Terre to Malabar or vice versa. It is therefore reasonable to assume that a direct route was taken for the tortoises en route to Picard, given that managing/transporting giant tortoises is a considerable effort. Eventually, more samples from both of these populations, as well as outgroups to quantify the magnitude of the flow and try to date it, are needed to confirm our hypothesis of across water gene flow. Evidence for ongoing gene flow over tens of generations would support our current hypothesis. The higher within‐island differentiation between M1 and M2 could be a result of the aforementioned flow to Malabar, but limited gene flow with M2. The vegetation between the regions is very dense *Pemphis* scrub with the exception of the coastal path, and a previous study on tortoise habitat use showed that Malabar tortoises, in general, have smaller home ranges compared to residents of other islands (Walton et al., 2019). However, it remains hard to explain why M1 looks genetically very similar to GS&W. Sampling the western part of Malabar and increasing numbers of samples throughout the atoll would likely shed light on this unexpected observation.

One potential reason for a lack of genetic differentiation between the GS&W and P is the movement of P tortoises to GW via the wide channel and islets between the two islands (Figure 2a), so that admixed P individuals influenced the GW group. However, this genetic similarity is likely to be driven by the founding history of the Picard population, which received individuals from Grande Terre (Bourn et al., 1999).

In summary, our findings suggest a subtle and unexpected signal of east–west population structure in *A*. *gigantea*, mainly correlated with landscape features, distance, as well as human‐induced reintroductions (primarily on Picard). Seawater may play a less important role as a barrier than has been previously assumed (Balmer et al., 2011; Grubb, 1971), instead water currents may support movements. Balmer et al. (2011) found no variation at the mitochondrial control region and there is currently no evidence for an ancient split into genetic groups. Given the very long generation time of giant tortoises, the substructure could still be several hundred years old and predate the species bottleneck.

### 4.2 Limitations of the study

Our study provides the first genomic insight on the wild Aldabra giant tortoise population and the number of tortoises per each main island of the atoll included in this study was limited to 11. Population structure analyses have shown to be robust to extremely low (e.g., 0.125×; Lou et al., 2021) and highly uneven per‐sample coverage (e.g., 0.5× to 6×; Skotte et al., 2013). But the sequencing effort (i.e., the combination of the number of samples and the sequencing depth per sample) affects the population genetic inferences obtained with genotype likelihood‐based allele frequency estimations (Buerkle & Gompert, 2013). In a recent review including experimental design recommendations for different types of population genomic analyses using low‐coverage whole‐genome sequencing data, it was suggested to prioritize the total number of samples (≥10 samples per population) over per‐sample coverage and to aim for ≥10× coverage per population both for population structure analyses (i.e., PCA and admixture analyses) and relative estimation of rare allele‐dependent metrics (i.e., pairwise *F*_ST_ and genetic diversity estimates). Our study design includes 11 samples (average coverage ~25×) per island (and originally expected population), and 5–6 samples (average coverage 11.4–13.7×) per sampling locality/genetic group. The unexpected outcome that there is more population structure than foreseen led to a rather low sample size per genetic group, while the coverage is still within the recommended range. Theoretically one would expect that these recommendations for whole‐genome sequencing would apply for the reduced representation sequencing approach as well, given that the latter is a representative of the former. Our study does indeed show that it is possible to find subtle population structure with this kind of data. Nevertheless, we believe future population genomics studies of Aldabra giant tortoises would highly benefit from more samples per locality and per population as well as more extensive geographical sampling, for example, further western parts of Malabar and small islands in the Aldabra Lagoon.

### 4.3 Conservation and research implications

Our study has several conservation implications. Specifically for the study species, our findings suggest that if giant tortoises from Aldabra are to be used for translocations, translocated individuals should ideally represent and potentially retain the overall genetic variation in the wild population. The local population on Picard was extinct and the current population is based on several bouts of turtle translocations since the early 1900s (Bourn et al., 1999) with the last occurring in the 1980s. Interestingly, individuals from Picard showed a mixture of the genetic assignments found on the other islands (Figure 2), suggesting that the translocations likely involved more than one source. This is in accordance with the relatively high genetic diversity. Since the island also hosts the research station, individuals taken from Picard could be a valuable alternative and logistically more feasible than trying to capture individuals across the entire atoll. However, our study does not yet give insights into the potential benefit of using captive or already rewilded populations for future translocations. Because *A*. *gigantea* were heavily exploited and exported to the outside of Aldabra Atoll in the 19th century (potentially before and during the species bottleneck) (Stoddart & Peake, 1979), it is not impossible that some of the original diversity now lost in the wild can still be found elsewhere. In any case, it is advised to translocate as many individuals as possible to minimize founder effects (Frankham et al., 2007). This should facilitate genetic management and monitoring of ongoing and future rewilding projects, including spatially larger projects in Madagascar (Pedrono et al., 2013), to maximize the evolutionary potential and survival of rewilded populations. As previously suggested by Balmer et al. (2011), we found no evidence for large differences in genetic diversity among the main islands and we currently do not see the need for translocations between islands. The similar diversity among islands also suggests that the observed population structure is unlikely to be explained by the species bottleneck, for instance, by much stronger reduction and then isolation of the eastern part of Grande Terre. However, we caution that our method of low‐coverage ddRAD has not been tested sufficiently for its reliability on the estimation of exact diversity measures (see recommendations from Lou et al., 2021).

Our study underlines the importance of genetically informed management decisions by showing unexpected population structure as previously discovered in Iberian wolves, Peruvian diving petrels, and Atlantic puffins, among others (Cristofari et al., 2019; Kersten et al., 2021; Silva et al., 2018). Considering that both reduced representation sequencing and low‐coverage approaches aim to decrease costs, our approach could be used in the population genomics of other vertebrates to address similar research questions. Our approach could be particularly suitable for systems with large genomes and no or only little genetic knowledge is available when aiming at a first overall look at population structure. Finally, our study shows that land‐ and seascape genetics should go hand in hand because terrestrial organisms living close to the sea could be influenced by both.

## CONFLICT OF INTEREST

The authors declare no conflict of interest.

## AUTHOR CONTRIBUTIONS

**F. Gözde Çilingir:** Formal analysis (lead); Funding acquisition (supporting); Writing – original draft (lead); Writing – review & editing (equal). **Dennis Hansen:** Resources (equal); Supervision (supporting); Writing – review & editing (supporting). **Nancy Bunbury:** Resources (equal); Writing – review & editing (supporting). **Erik Postma:** Conceptualization (supporting); Resources (supporting); Writing – review & editing (supporting). **Richard Baxter:** Resources (equal); Writing – review & editing (supporting). **Lindsay Turnbull:** Resources (supporting); Writing – review & editing (supporting). **Arpat Ozgul:** Funding acquisition (lead); Supervision (supporting); Writing – review & editing (supporting). **Christine Grossen:** Conceptualization (lead); Formal analysis (supporting); Funding acquisition (lead); Supervision (lead); Writing – original draft (supporting); Writing – review & editing (equal).

## Supporting information

Figures S1‐S6

Table S1

Table S2

## Data Availability

The sequencing data that support the findings of this study are available at the NCBI Sequence Read Archive with the accession numbers SRX10954672–SRX10954704.

## References

[ece38739-bib-0001] Andrews, K. R. , Good, J. M. , Miller, M. R. , Luikart, G. , & Hohenlohe, P. A. (2016). Harnessing the power of RADseq for ecological and evolutionary genomics. Nature Reviews Genetics, 17(2), 81–92. 10.1038/nrg.2015.28 PMC4823021 26729255

[ece38739-bib-0002] Andrews, S. (2010). FastQC: A quality control tool for high throughput sequence data. http://www.bioinformatics.babraham.ac.uk/projects/fastqc/

[ece38739-bib-0003] Arjona, Y. , Fernández‐López, J. , Navascués, M. , Alvarez, N. , Nogales, M. , & Vargas, P. (2020). Linking seascape with landscape genetics: Oceanic currents favour colonization across the Galápagos Islands by a coastal plant. Journal of Biogeography, 47(12), 2622–2633. 10.1111/jbi.13967

[ece38739-bib-0004] Austin, J. J. , Nicholas Arnold, E. , & Bour, R. (2003). Was there a second adaptive radiation of giant tortoises in the Indian Ocean? Using mitochondrial DNA to investigate speciation and biogeography of Aldabrachelys (Reptilia, Testudinidae). Molecular Ecology, 12(6), 1415–1424. 10.1046/j.1365-294X.2003.01842.x 12755871

[ece38739-bib-0005] Balmer, O. , Ciofi, C. , Galbraith, D. A. , Swingland, I. R. , Zug, G. R. , & Caccone, A. (2011). Population genetic structure of Aldabra giant tortoises. The Journal of Heredity, 102(1), 29–37. 10.1093/jhered/esq096 20805288

[ece38739-bib-0006] Bay, R. A. , Taylor, E. B. , & Schluter, D. (2019). Parallel introgression and selection on introduced alleles in a native species. Molecular Ecology, 28, 2802–2813. 10.1111/mec.15097 30980778

[ece38739-bib-0007] Bolger, A. M. , Lohse, M. , & Usadel, B. (2014). Trimmomatic: a flexible trimmer for Illumina sequence data. Bioinformatics, 30(15), 2114–2120. 10.1093/bioinformatics/btu170 24695404 PMC4103590

[ece38739-bib-0008] Bourn, D. , & Coe, M. (1978). The size, structure and distribution of the giant tortoise population of Aldabra. Philosophical Transactions of the Royal Society of London. Series B: Biological Sciences, 282(988), 139–175. 10.1098/rstb.1978.0011

[ece38739-bib-0009] Bourn, D. , Gibson, C. , Augeri, D. , Wilson, C. J. , Church, J. , & Hay, S. I. (1999). The rise and fall of the Aldabran giant tortoise population. Proceedings of the Royal Society B: Biological Sciences, 266(1424), 1091–1100. 10.1098/rspb.1999.0748 PMC1689958 10406128

[ece38739-bib-0010] Breusing, C. , Johnson, S. B. , Vrijenhoek, R. C. , & Young, C. R. (2019). Host hybridization as a potential mechanism of lateral symbiont transfer in deep‐sea vesicomyid clams. Molecular Ecology, 28(21), 4697–4708. 10.1111/mec.15224 31478269 PMC7004080

[ece38739-bib-0011] Buerkle, A. C. , & Gompert, Z. (2013). Population genomics based on low coverage sequencing: how low should we go? Molecular Ecology, 22(11), 3028–3035. 10.1111/mec.12105 23174005

[ece38739-bib-0012] Bunbury, N. , von Brandis, R. , Currie, J. C. , van de Crommenacker, J. , Accouche, W. , Birch, D. , Chong‐Seng, L. , Doak, N. , Haupt, P. , Haverson, P. , Jean‐Baptiste, M. , & Fleischer‐Dogley, F. (2018). Late stage dynamics of a successful feral goat eradication from the UNESCO World Heritage site of Aldabra Atoll, Seychelles. Biological Invasions, 20(7), 1735–1747. 10.1007/s10530-017-1657-0

[ece38739-bib-0013] Calsbeek, R. , & Smith, T. B. (2003). Ocean currents mediate evolution in island lizards. Nature, 426(6966), 552–555. 10.1038/nature02143 14654839

[ece38739-bib-0014] Carreras, C. , Ordóñez, V. , Zane, L. , Kruschel, C. , Nasto, I. , Macpherson, E. , & Pascual, M. (2017). Population genomics of an endemic Mediterranean fish: Differentiation by fine scale dispersal and adaptation. Scientific Reports, 7(1), 1–12. 10.1038/srep43417 28262802 PMC5338269

[ece38739-bib-0015] Çilingir, F. G. , Hansen, D. , Ozgul, A. , & Grossen, C. (2021). Design of SNP markers for Aldabra giant tortoises using low coverage ddRAD‐seq. Conservation Genetics Resources, 13(4), 409–412. 10.1007/s12686-021-01225-4

[ece38739-bib-0016] Çilingir, F. G. , Seah, A. , Horne, B. D. , Som, S. , Bickford, D. P. , & Rheindt, F. E. (2019). Last exit before the brink: Conservation genomics of the Cambodian population of the critically endangered southern river terrapin. Ecology and Evolution, 9(17), 9500–9510. 31534671 10.1002/ece3.5434 PMC6745661

[ece38739-bib-0017] Corlett, R. T. (2017). A bigger toolbox: biotechnology in biodiversity conservation. Trends in Biotechnology, 35(1), 55–65. 10.1016/j.tibtech.2016.06.009 27424151

[ece38739-bib-0018] Cristofari, R. , Plaza, P. , Fernández, C. E. , Trucchi, E. , Gouin, N. , Le Bohec, C. , Zavalga, C. , Alfaro‐Shigueto, J. , & Luna‐Jorquera, J. G. (2019). Unexpected population fragmentation in an endangered seabird: The case of the Peruvian diving‐petrel. Scientific Reports, 9(1), 2021. 10.1038/s41598-019-38682-9 30765805 PMC6375911

[ece38739-bib-0019] Davey, J. W. , & Blaxter, M. L. (2010). RADSeq: next‐generation population genetics. Briefings in Functional Genomics, 9(5–6), 416–423. 10.1093/bfgp/elq031 21266344 PMC3080771

[ece38739-bib-0020] Davey, J. W. , Cezard, T. , Fuentes‐Utrilla, P. , Eland, C. , Gharbi, K. , & Blaxter, M. L. (2013). Special features of RAD Sequencing data: implications for genotyping. Molecular Ecology, 22(11), 3151–3164. 23110438 10.1111/mec.12084 PMC3712469

[ece38739-bib-0021] David, F. N. , & Tukey, J. W. (1977). Exploratory data analysis. Biometrics, 33(4), 768. 10.2307/2529486

[ece38739-bib-0022] Delport, W. , Grant, T. J. , Ryan, P. G. , & Bloomer, P. (2006). Ten microsatellite loci for evolutionary research on Nesospiza buntings. Molecular Ecology Notes, 6(4), 1180–1183. 10.1111/j.1471-8286.2006.01485.x

[ece38739-bib-0023] Ellegren, H. , Primmer, C. R. , & Sheldon, B. C. (1995). Microsatellite “evolution”: Directionality or bias? Nature Genetics, 11(4), 360–362. 10.1038/ng1295-360 7493011

[ece38739-bib-0024] Fox, E. A. , Wright, A. E. , Fumagalli, M. , & Vieira, F. G. (2019). ngsLD: Evaluating linkage disequilibrium using genotype likelihoods. Bioinformatics, 35(19), 3855–3856. 10.1093/bioinformatics/btz200 30903149

[ece38739-bib-0025] Frankham, R. , Ballou, S. E. J. , Briscoe, D. A. , & Ballou, J. D. (2007). Introduction to conservation genetics. Cambridge University Press.

[ece38739-bib-0026] Fumagalli, M. , Vieira, F. G. , Linderoth, T. , & Nielsen, R. (2014). ngsTools: methods for population genetics analyses from next‐generation sequencing data. Bioinformatics, 30(10), 1486–1487. 10.1093/bioinformatics/btu041 24458950 PMC4016704

[ece38739-bib-0027] Gibson, C. W. D. , & Hamilton, J. (1984). Population processes in a large herbivorous reptile: The giant tortoise of Aldabra Atoll. Oecologia, 61(2), 230–240. 10.1007/BF00396766 28309417

[ece38739-bib-0028] Griffiths, C. J. , Hansen, D. M. , Jones, C. G. , Zuël, N. , & Harris, S. (2011). Resurrecting extinct interactions with extant substitutes. Current Biology, 21(9), 762–765. 10.1016/j.cub.2011.03.042 21514155

[ece38739-bib-0029] Griffiths, C. J. , Zuël, N. , Jones, C. G. , Ahamud, Z. , & Harris, S. (2013). Assessing the potential to restore historic grazing ecosystems with tortoise ecological replacements. Conservation Biology, 27(4), 690–700. 10.1111/cobi.12087 23773124

[ece38739-bib-0030] Grubb, P. (1971). The growth, ecology and population structure of giant tortoises on Aldabra. Philosophical Transactions of the Royal Society of London. Series B: Biological Sciences, 260(836), 327–372. 10.1098/rstb.1971.0018

[ece38739-bib-0031] Hanghøj, K. , Moltke, I. , Andersen, P. A. , Manica, A. , & Korneliussen, T. S. (2019). Fast and accurate relatedness estimation from high‐throughput sequencing data in the presence of inbreeding. GigaScience, 8(5), giz034. 10.1093/gigascience/giz034 31042285 PMC6488770

[ece38739-bib-0032] Hansen, D. M. (2015). Non‐native megaherbivores: The case for novel function to manage plant invasions on islands. AoB Plants, 7, lv085. 10.1093/aobpla/plv085 PMC4565891 26194166

[ece38739-bib-0033] Hansen, D. M. , Austin, J. J. , Baxter, R. H. , de Boer, E. J. , Falcón, W. , Norder, S. J. , Rijsdijk, K. F. , Thébaud, C. , Bunbury, N. , & Warren, B. H. (2017). Origins of endemic island tortoises in the western Indian Ocean: A critique of the human‐translocation hypothesis. Journal of Biogeography, 44, 1430–1435. 10.1111/jbi.12893

[ece38739-bib-0034] Hansen, D. M. , Josh Donlan, C. , Griffiths, C. J. , & Campbell, K. J. (2010). Ecological history and latent conservation potential: Large and giant tortoises as a model for taxon substitutions. Ecography, 33, 272–284. 10.1111/j.1600-0587.2010.06305.x

[ece38739-bib-0035] Hardy, G. H. (1908). Mendelian proportions in a mixed population. Science, 28(706), 49–50. 10.1126/science.28.706.49 17779291

[ece38739-bib-0036] Hawlitschek, O. , Ramírez Garrido, S. , & Glaw, F. (2017). How marine currents influenced the widespread natural overseas dispersal of reptiles in the Western Indian Ocean region. Journal of Biogeography, 44(6), 1435–1440. 10.1111/jbi.12940

[ece38739-bib-0037] Hayden, E. C. (2014). Technology: The $1,000 genome. Nature, 507(7492), 294–295. 24646979 10.1038/507294a

[ece38739-bib-0038] Hendricks, S. , Epstein, B. , Schönfeld, B. , Wiench, C. , Hamede, R. , Jones, M. , Storfer, A. , & Hohenlohe, P. (2017). Conservation implications of limited genetic diversity and population structure in Tasmanian devils (*Sarcophilus harrisii*). Conservation Genetics, 18(4), 977–982. 10.1007/s10592-017-0939-5 28966567 PMC5614444

[ece38739-bib-0039] Hnatiuk, R. J. , Woodell, S. R. J. , & Bourn, D. M. (1976). Giant tortoise and vegetation interactions on Aldabra atoll—part 2: Coastal. Biological Conservation, 9(4), 305–316. 10.1016/0006-3207(76)90052-5

[ece38739-bib-0040] Hoban, S. , Bruford, M. W. , Chris Funk, W. , Galbusera, P. , Patrick Griffith, M. , Grueber, C. E. , Heuertz, M. , Hunter, M. E. , Hvilsom, C. , Stroil, B. K. , Kershaw, F. , Khoury, C. K. , Laikre, L. , Lopes‐Fernandes, M. , MacDonald, A. J. , Mergeay, J. , Meek, M. , Mittan, C. , Mukassabi, T. A. , … Vernesi, C. (2021). Global commitments to conserving and monitoring genetic diversity are now necessary and feasible. BioScience, 71(9), 964–976. 10.1093/biosci/biab054 34475806 PMC8407967

[ece38739-bib-0041] Hohenlohe, P. A. , Amish, S. J. , Catchen, J. M. , Allendorf, F. W. , & Luikart, G. (2011). Next‐generation RAD sequencing identifies thousands of SNPs for assessing hybridization between rainbow and westslope cutthroat trout. Molecular Ecology Resources, 11, 117–122. 10.1111/j.1755-0998.2010.02967.x 21429168

[ece38739-bib-0042] Jacquard, A. (1974). The genetic structure of populations. Springer Science & Business Media.

[ece38739-bib-0043] Kehlmaier, C. , Graciá, E. , Campbell, P. D. , Hofmeyr, M. D. , Schweiger, S. , Martínez‐Silvestre, A. , Joyce, W. , & Fritz, U. (2019). Ancient mitogenomics clarifies radiation of extinct Mascarene giant tortoises (*Cylindraspis* spp.). Scientific Reports, 9(1), 17487. 10.1038/s41598-019-54019-y 31767921 PMC6877638

[ece38739-bib-0044] Kersten, O. , Star, B. , Leigh, D. M. , Anker‐Nilssen, T. , Strøm, H. , Danielsen, H. S. J. , Descamps, S. , Erikstad, K. E. , Fitzsimmons, M. G. , Fort, J. , Hansen, E. J. , Harris, M. P. , Irestedt, M. , Kleven, O. , Mallory, M. L. , Jakobsen, K. S. , & Boessenkool, S. (2021). Complex population structure of the Atlantic puffin revealed by whole genome analyses. Communications Biology, 4(1), 922. 10.1038/s42003-021-02415-4 34326442 PMC8322098

[ece38739-bib-0045] Kopelman, N. M. , Mayzel, J. , Jakobsson, M. , Rosenberg, N. A. , & Mayrose, I. (2015). Clumpak: a program for identifying clustering modes and packaging population structure inferences across K. Molecular Ecology Resources, 15(5), 1179–1191. 25684545 10.1111/1755-0998.12387 PMC4534335

[ece38739-bib-0046] Korneliussen, T. S. , Albrechtsen, A. , & Nielsen, R. (2014). ANGSD: Analysis of next generation sequencing data. BMC Bioinformatics, 15, 356. 10.1186/s12859-014-0356-4 25420514 PMC4248462

[ece38739-bib-0047] Lepais, O. , & Weir, J. T. (2014). SimRAD: an R package for simulation‐based prediction of the number of loci expected in RADseq and similar genotyping by sequencing approaches. Molecular Ecology Resources, 14(6), 1314–1321. 10.1111/1755-0998.12273 24806844

[ece38739-bib-0048] Li, H. (2011). Improving SNP discovery by base alignment quality. Bioinformatics, 27(8), 1157–1158. 10.1093/bioinformatics/btr076 21320865 PMC3072548

[ece38739-bib-0049] Li, H. , & Durbin, R. (2009). Fast and accurate short read alignment with Burrows‐Wheeler transform. Bioinformatics, 25(14), 1754–1760. 10.1093/bioinformatics/btp324 19451168 PMC2705234

[ece38739-bib-0050] Li, H. , Handsaker, B. , Wysoker, A. , Fennell, T. , Ruan, J. , Homer, N. , Marth, G. , Abecasis, G. , Durbin, R. , & 1000 Genome Project Data Processing Subgroup (2009). The sequence alignment/map format and SAMtools. Bioinformatics, 25(16), 2078–2079. 10.1093/bioinformatics/btp352 19505943 PMC2723002

[ece38739-bib-0051] Lou, R. N. , Jacobs, A. , Wilder, A. , & Therkildsen, N. O. (2021). A beginner’s guide to low‐coverage whole genome sequencing for population genomics. Molecular Ecology Resources, 30, 5966–5993. 10.1111/mec.16077 34250668

[ece38739-bib-0052] Mesnick, S. L. , Taylor, B. L. , Archer, F. I. , Martien, K. K. , Treviño, S. E. , Hancock‐Hanser, B. L. , Moreno Medina, S. C. , Pease, V. L. , Robertson, K. M. , Straley, J. M. , Baird, R. W. , Calambokidis, J. , Schorr, G. S. , Wade, P. , Burkanov, V. , Lunsford, C. R. , Rendell, L. , & Morin, P. A. (2011). Sperm whale population structure in the eastern and central North Pacific inferred by the use of singlenucleotide polymorphisms, microsatellites and mitochondrial DNA. Molecular Ecology Resources, 11, 278–298. 10.1111/j.1755-0998.2010.02973.x 21429181

[ece38739-bib-0053] McKenna, A. , Hanna, M. , Banks, E. , Sivachenko, A. , Cibulskis, K. , Kernytsky, A. , Garimella, K. , Altshuler, D. , Gabriel, S. , Daly, M. , & DePristo, M. A. (2010). The genome analysis toolkit: a MapReduce framework for analyzing next‐generation DNA sequencing data. Genome Research, 20(9), 1297–1303. 10.1101/gr.107524.110 20644199 PMC2928508

[ece38739-bib-0054] Meisner, J. , & Albrechtsen, A. (2018). Inferring population structure and admixture proportions in low‐depth NGS data. Genetics, 210(2), 719–731. 10.1534/genetics.118.301336 30131346 PMC6216594

[ece38739-bib-0055] Merton, L. F. H. , Bourn, D. M. , & Hnatiuk, R. J. (1976). Giant tortoise and vegetation interactions on aldabra atoll—part 1: Inland. Biological Conservation, 9(4), 293–304. 10.1016/0006-3207(76)90051-3

[ece38739-bib-0056] O’Leary, S. J. , Puritz, J. B. , Willis, S. C. , Hollenbeck, C. M. , & Portnoy, D. S. (2018). These aren’t the loci you're looking for: Principles of effective SNP filtering for molecular ecologists. Molecular Ecology, 27, 3193–3206. 10.1111/mec.14792 29987880

[ece38739-bib-0057] Palkovacs, E. P. , Gerlach, J. , & Caccone, A. (2002). The evolutionary origin of Indian Ocean tortoises (Dipsochelys). Molecular Phylogenetics and Evolution, 24(2), 216–227. 10.1016/S1055-7903(02)00211-7 12144758

[ece38739-bib-0058] Pasaniuc, B. , Rohland, N. , McLaren, P. J. , Garimella, K. , Zaitlen, N. , Li, H. , Gupta, N. , Neale, B. M. , Daly, M. J. , Sklar, P. , Sullivan, P. F. , Bergen, S. , Moran, J. L. , Hultman, C. M. , Lichtenstein, P. , Magnusson, P. , Purcell, S. M. , Haas, D. W. , Liang, L. , … Price, A. L. (2012). Extremely low‐coverage sequencing and imputation increases power for genome‐wide association studies. Nature Genetics, 44(6), 631–635. 10.1038/ng.2283 22610117 PMC3400344

[ece38739-bib-0059] Peart, C. R. , Tusso, S. , Pophaly, S. D. , Botero‐Castro, F. , Wu, C.‐C. , Aurioles‐Gamboa, D. , Baird, A. B. , Bickham, J. W. , Fircada, J. , Galimberti, F. , Gemmell, N. J. , Hoffman, J. I. , Kovacs, K. M. , Kunnasranta, M. , Lydersen, C. , Nyman, T. , de Oliveira, L. R. , Orr, A. J. , Sanvito, S. , … Wolf, J. B. W. (2020). Determinants of genetic variation across eco‐evolutionary scales in pinnipeds. Nature Ecology & Evolution, 4(8), 1095–1104. 10.1038/s41559-020-1215-5 32514167

[ece38739-bib-0060] Pedrono, M. , Griffiths, O. L. , Clausen, A. , Smith, L. L. , Griffiths, C. J. , Wilmé, L. , & Burney, D. A. (2013). Using a surviving lineage of Madagascar’s vanished megafauna for ecological restoration. Biological Conservation, 159, 501–506. 10.1016/j.biocon.2012.11.027

[ece38739-bib-0061] Peterson, B. K. , Weber, J. N. , Kay, E. H. , Fisher, H. S. , & Hoekstra, H. E. (2012). Double digest RADseq: An inexpensive method for de novo SNP discovery and genotyping in model and non‐model species. PLoS One, 7(5), e37135. 10.1371/journal.pone.0037135 22675423 PMC3365034

[ece38739-bib-0062] Pritchard, J. K. , Stephens, M. , & Donnelly, P. (2000). Inference of population structure using multilocus genotype data. Genetics, 155(2), 945–959. 10.1093/genetics/155.2.945 10835412 PMC1461096

[ece38739-bib-0063] Quesada, V. , Freitas‐Rodríguez, S. , Miller, J. , Pérez‐Silva, J. G. , Jiang, Z.‐F. , Tapia, W. , Santiago‐Fernández, O. , Campos‐Iglesias, D. , Kuderna, L. F. K. , Quinzin, M. , Álvarez, M. G. , Carrero, D. , Beheregaray, L. B. , Gibbs, J. P. , Chiari, Y. , Glaberman, S. , Ciofi, C. , Araujo‐Voces, M. , Mayoral, P. , … López‐Otín, C. (2019). Giant tortoise genomes provide insights into longevity and age‐related disease. Nature Ecology & Evolution, 3(1), 87–95. 10.1038/s41559-018-0733-x 30510174 PMC6314442

[ece38739-bib-0064] Quinlan, A. R. , & Hall, I. M. (2010). BEDTools: A flexible suite of utilities for comparing genomic features. Bioinformatics, 26(6), 841–842. 10.1093/bioinformatics/btq033 20110278 PMC2832824

[ece38739-bib-0065] R Core Team (2020). R: A language and environment for statistical computing. R Foundation for Statistical Computing.

[ece38739-bib-0066] Reed, D. H. (2005). Relationship between population size and fitness. Conservation Biology, 19(2), 563–568. 10.1111/j.1523-1739.2005.00444.x

[ece38739-bib-0067] Reed, D. H. , & Frankham, R. (2003). Correlation between fitness and genetic diversity. Conservation Biology, 17(1), 230–237. 10.1046/j.1523-1739.2003.01236.x

[ece38739-bib-0068] Rochette, N. C. , Rivera‐Colón, A. G. , & Catchen, J. M. (2019). Stacks 2: Analytical methods for paired‐end sequencing improve RADseq‐based population genomics. Molecular Ecology, 28, 4737–4754. 10.1111/mec.15253 31550391

[ece38739-bib-0069] Shafer, A. B. A. , Wolf, J. B. W. , Alves, P. C. , Bergström, L. , Bruford, M. W. , Brännström, I. , Bruford, M. W. , Brännström, I. , Colling, G. , Dalén, L. , Meester, L. D. , Ekblom, R. , Fawcett, K. D. , Fior, S. , Hajibabaei, M. , Hill, J. A. , Hoezel, A. R. , Höglund, J. , Jensen, E. L. , … Zieliński, P. (2015). Genomics and the challenging translation into conservation practice. Trends in Ecology & Evolution, 30(2), 78–87. 10.1016/j.tree.2014.11.009 25534246

[ece38739-bib-0070] Silva, P. , López‐Bao, J. V. , Llaneza, L. , Álvares, F. , Lopes, S. , Blanco, J. C. , Cortés, Y. , García, E. , Palacios, V. , Rio‐Maior, H. , Ferrand, N. , & Godinho, R. (2018). Cryptic population structure reveals low dispersal in Iberian wolves. Scientific Reports, 8(1), 14108. 10.1038/s41598-018-32369-3 30237419 PMC6147861

[ece38739-bib-0071] Skotte, L. , Korneliussen, T. S. , & Albrechtsen, A. (2013). Estimating individual admixture proportions from next generation sequencing data. Genetics, 195(3), 693–702. 10.1534/genetics.113.154138 24026093 PMC3813857

[ece38739-bib-0072] Stoddart, D. R. , & Peake, J. F. (1979). Historical records of Indian Ocean giant tortoise populations. Philosophical Transactions of the Royal Society of London. Series B: Biological Sciences, 286, 147–161.

[ece38739-bib-0073] Swingland, I. R. , North, P. M. , Dennis, A. , & Parker, M. J. (1989). Movement patterns and morphometrics in giant tortoises. The Journal of Animal Ecology, 58(3), 971–985. 10.2307/5136

[ece38739-bib-0074] Tajima, F. (1989). Statistical method for testing the neutral mutation hypothesis by DNA polymorphism. Genetics, 123(3), 585–595. 10.1093/genetics/123.3.585 2513255 PMC1203831

[ece38739-bib-0075] Turnbull, L. A. , Ozgul, A. , Accouche, W. , Baxter, R. , Chong Seng, L. , Currie, J. C. , Doak, N. , Hansen, D. M. , Pistorius, P. , Richards, H. , van de Crommenacker, J. , von Brandis, R. , Fleischer‐Dogley, F. , & Bunbury, N. (2015). Persistence of distinctive morphotypes in the native range of the CITES‐listed Aldabra giant tortoise. Ecology and Evolution, 5(23), 5499–5508. 10.1002/ece3.1764 27069601 PMC4813117

[ece38739-bib-0076] Turtle Taxonomy Working Group [Rhodin, A.G.J., Iverson, J.B., Bour, R., Fritz, U., Georges, A., Shaffer, H.B., and van Dijk, P.P.] (2017). Turtles of the World: Annotated Checklist and Atlas of Taxonomy, Synonymy, Distribution, and Conservation Status (8th Ed.): Conservation Biology of Freshwater Turtles and Tortoises: a Compilation Project of the IUCN/SSC Tortoise and Freshwater Turtle Specialist Group.

[ece38739-bib-0077] Walton, R. , Baxter, R. , Bunbury, N. , Hansen, D. , Fleischer‐Dogley, F. , Greenwood, S. , & Schaepman‐Strub, G. (2019). In the land of giants: habitat use and selection of the Aldabra giant tortoise on Aldabra Atoll. Biodiversity and Conservation, 28, 3183–3198. 10.1007/s10531-019-01813-9

[ece38739-bib-0078] Warmuth, V. M. , & Ellegren, H. (2019). Genotype‐free estimation of allele frequencies reduces bias and improves demographic inference from RADSeq data. Molecular Ecology Resources, 19(3), 586–596. 10.1111/1755-0998.12990 30633448

[ece38739-bib-0079] Watterson, G. A. (1975). On the number of segregating sites in genetical models without recombination. Theoretical Population Biology, 7(2), 256–276. 10.1016/0040-5809(75)90020-9 1145509

[ece38739-bib-0080] White, C. , Selkoe, K. A. , Watson, J. , Siegel, D. A. , Zacherl, D. C. , & Toonen, R. J. (2010). Ocean currents help explain population genetic structure. Proceedings of the Royal Society B: Biological Sciences, 277(1688), 1685–1694. 10.1098/rspb.2009.2214 PMC2871860 20133354

[ece38739-bib-0081] Wickham, H. (2016). ggplot2: Elegant graphics for data analysis. https://ggplot2.tidyverse.org

[ece38739-bib-0082] Wietlisbach, X. (2017). The genetics of giants: how have time and space shaped genetic variation within and among Aldabra giant tortoise populations? (MSc.; E. Postma, Ed.). University of Zurich.

[ece38739-bib-0083] Záveská, E. , Maylandt, C. , Paun, O. , Bertel, C. , Frajman, B. , The Steppe Consortium , & Schönswetter, P. (2019). Multiple auto‐ and allopolyploidisations marked the Pleistocene history of the widespread Eurasian steppe plant Astragalus onobrychis (Fabaceae). Molecular Phylogenetics and Evolution, 139, 106572. 10.1016/j.ympev.2019.106572 31351183

